# Unraveling Changes of Brachial Artery Residual Stress and Its Relationship to Cardiovascular Disease Risk Factors

**DOI:** 10.31083/j.rcm2508289

**Published:** 2024-08-16

**Authors:** Jianxiong Chen, Lin Jin, Lei Sha, Mengmeng Cao, Lianfang Du, Zhaojun Li, Xianghong Luo

**Affiliations:** ^1^Department of Ultrasound, Shanghai General Hospital of Nanjing Medical University, 200080 Shanghai, China; ^2^Department of Ultrasound, Mindong Hospital Affiliated to Fujian Medical University, 355000 Ningde, Fujian, China; ^3^Department of Ultrasound, Guanghua Hospital Affiliated to Shanghai University of Traditional Chinese Medicine, 200052 Shanghai, China; ^4^Department of Ultrasound, Shanghai General Hospital Jiading Branch, Shanghai Jiaotong University School of Medicine, 200080 Shanghai, China; ^5^Department of Ultrasound, The Shanghai General Hospital, Shanghai Jiaotong University School of Medicine, 200080 Shanghai, China; ^6^Department of Echocardiography, The Shanghai General Hospital, Shanghai Jiaotong University School of Medicine, 200080 Shanghai, China

**Keywords:** arterial stiffness, brachial artery, natural population, residual stress, aging

## Abstract

**Background:**

Arterial pressure volume index (API) offers a non-invasive measurement of brachial artery residual stress. This study investigated API distribution characteristics and correlations with cardiovascular disease risk (CVD) factors in a large Chinese population sample.

**Methods:**

This cross-sectional study surveyed a total of 7620 participants. We analyzed the relationships between API and factors influencing CVD, using regression-based stepwise backward selection and restrictive cubic spline models to express relationships as standardized beta values.

**Results:**

Multiple linear regression analysis identified many independent factors influencing API including age, sex, body mass index (BMI), pulse pressure (PP), heart rate (HR), hemoglobin, uric acid (UA), estimated glomerular filtration rate (eGFR), triglyceride (TC), and a history of hypertension. Notably, API values increased at 33 and escalated with advancing age. Increases in API were associated with rises in PP and UA increases, particularly when PP reached 60 mmHg and the UA reached 525 units. Conversely, API was found to decrease with elevated HR and eGFR. Furthermore, there was a significant inverted U-shaped relationship between API and BMI.

**Conclusions:**

This study was the first to describe API distribution characteristics in a large sample of the Chinese population, providing references for evaluating API changes in the assessment of residual stress variations in diverse diseases. Notably, API displayed a U-shaped relationship with age and was closely related to traditional CVD risk factors, underscoring its potential as a non-invasive tool for risk assessment in vascular health.

**Clinical Trial Registration:**

This research was registered with the China Clinical Trial Registration Center (Registration Number: ChiCTR2000035937).

## 1. Introduction

Residual stress, defined as stress within blood vessels in a no-load state (after blood pressure removal) [1, 2], and plays a crucial role in maintaining the normal physiological function of various organs [3, 4]. Previous studies have highlighted its role in vascular tissue growth, development, and the progression of pathologies including aneurysms [5, 6]. Moreover, it significantly impacts the peak cap stress in vulnerable arterial plaques, thereby serving as a predictor for plaque rupture risk—a leading cause of acute cardiovascular events [7]. Therefore, residual stress serves as a valuable indicator of arterial stiffness [5]. Despite its significance, measuring arterial residual stress remains challenging since it is primarily obtained through biomechanical experimental studies [8]. Consequently, there’s an urgent need to develop new, rapid, convenient, and non-invasive vascular residual stress indicators.

Recent studies have shown that the arterial pressure-volume index (API), obtained by analysis of upper arm cuff oscillations, reflects the residual circumferential stress in peripheral arteries at a zero-stress state [9]. Given that higher API values indicate greater arterial wall stiffness, it can be calculated as the reciprocal value of the slope of the sigmoidal curve, reflecting the relationship between cuff pressure and arterial volume, offering a non-invasive gauge to assess muscular arterial stiffness [10, 11]. Prior cross-sectional studies found that API was significantly associated with carotid arterial compliance, pulse wave velocity (PWV), as well as arterial stiffness in the general population [12, 13]. Sasaki-Nakashima *et al*. [14] found that API was significantly related to the Framingham cardiovascular risk score and the Suita score, which independently predict cardiovascular disease. Furthermore, Ueda *et al*. [15] reported significant coronary stenosis as an independent determinant of API.

Currently, residual stress assessment by pressure oscillation waves is in early stages and has not yet been integrated into clinical practice. In addition, the distribution characteristics of API in Chinese populations have not been widely studied. Therefore, we aim to elucidate the distribution characteristics and influencing factors of API, alongside its variations and associations with cardiovascular disease (CVD) risk factors. By doing so, API may provide a new perspective for non-invasive assessment of residual stress related to cardiovascular diseases.

## 2. Materials and Methods

### 2.1 Study Participants

We screened 18,000 healthy participants for this study between August 2020 to June 2022. Each underwent brachial artery pressure measurements during an annual medical checkup at a single medical institution in Shanghai, China. The research protocol received ethical approval from the Ethics Committee of Shanghai General Hospital (Approval Number: 2019KY009-4) and was registered with the China Clinical Trial Registration Center (Registration Number: ChiCTR2000035937). In adherence to the stringent ethical standards as outlined in the Declaration of Helsinki, all participants willingly provided their informed consent.

### 2.2 Inclusion and Exclusion Criteria

The following inclusion criteria were used: (1) participants were 18 years or older and (2) demonstrated voluntarily participation in the study by signing an informed consent form. The exclusion criteria included (1) participants with a history of significant cardiovascular disease or vascular pathology affecting the extremities. This included participants with a history of angina, myocardial infarction, cerebrovascular accidents, or peripheral arterial disease, as well as those suffering from chronic kidney disease. (2) Participants with prior vascular interventions or limb amputations that interfered with accurate API measurement. (3) Those unable to provide the necessary cooperation for the completion of the measurements were also excluded.

### 2.3 Baseline Measurements

On the day of the examination, all participants abstained from antihypertensive medications, tobacco, alcohol, and caffeine consumption for a period of 24 hours prior to the study. Each participant was requested to complete an electronic survey, which captured essential demographic and health-related data, such as age, sex, smoking, alcohol consumption, their medical history including anti-hypertensive and anti-diabetic medication. Weight and height were measured by nurses according to standardized measurement protocols. The body mass index (BMI) was computed using the formula weight divided by the square of height (kg/m^2^).

### 2.4 Arterial Stiffness Indices and Blood Pressure

Measurements were based on previous study methods [10]. The participants were examined in a relaxed atmosphere at a controlled temperature. After 5 minutes resting in the sitting position, a portable arterial wave detector (PASESA AVE-2000Pro, Shisei Datum, Tokyo, Japan) was used to measure the values of brachial API, systolic blood pressure (SBP), diastolic blood pressure (DBP) and heart rate. Pulse pressure (PP) was calculated as follows: PP = SBP – DBP. The averages of two repeated measurements were recorded for the analysis. The measurements were taken 5 minutes apart to avoid reactive hyperemia.

The API was calculated as follows: An inverse tangent function (Eqn. 1) or S-type function (Eqn. 2) was used to fit the transmural pressure-vascular volume curve.

(1) Inverse tangent function: ⁢F⁢(χ)=Aarctan⁡(B⁢χ+C)+D

(2) $$\text{ S-type function: }\,G\,\left(\chi \right)=\frac{A}{1+e^{-B\,\chi +C}}+D$$

In the above functions, χ represents the transmural pressure to be fitted; A, B, C and D are the coefficients of the fitting function. API is defined as A⁢P⁢I=1/B, where X is a constant herein taken to be 1.

### 2.5 Blood Biochemical Analysis

Utilizing standardized reagents and a state-of-the-art automatic biochemical analyzer, we obtained the concentrations of total cholesterol (TC), high-density lipoprotein (HDL) cholesterol, low-density lipoprotein (LDL) cholesterol, fasting blood glucose (FBG), uric acid (UA), aspartate aminotransferase (AST), and the estimated glomerular filtration rate (eGFR). To ensure the integrity of the samples, the blood samples were subjected to centrifugation at a vigorous pace of 8000 revolutions per minute for a duration of 12 minutes and stored under ultra-low temperatures of –80 °C for subsequent analysis.

### 2.6 Reproducibility Analysis

Using a stratified random sampling approach, partitioned by age and sex, 300 participants were enrolled to evaluate the inter- and intra-group measurement reliability. API values were independently measured by experienced nurses, followed by a repeat assessment by the initial nurse after a seven-day interval, thereby verifying the consistency and dependability of the obtained results.

### 2.7 Statistical Analysis

In the descriptive analysis, continuous variables were presented as mean ± standard deviation (SD). Categorical variables were analyzed with the Chi-square test and continuous variables with one-way analysis of variance (ANOVA), and post-hoc Least Significant Difference (LSD) test for evaluating differences among 3 groups. Multivariable regression analysis was employed to examine the correlations between API and its influencing factors. The model construction involved a rigorous backward stepwise approach, incorporating baseline characteristics, and maintaining a stringent significance threshold of 0.1. Variables achieving this benchmark in any of the iterations were integrated into the final comprehensive multivariable model. We computed standardized β coefficients, which quantitatively express the degree to which a dependent variable is likely to alter in response to a unit change of one standard deviation in the independent variable. This method facilitates the comparative assessment of the magnitude of associations among variables. Pearson’s correlation was used for correlation analyses.

The linear regression was used to obtain the independent influencing factors of the API. High API was defined as an API value equal to or greater than 31 [16]. The backward stepwise regression method was performed to further analyze the independent risk factors associated with a high API. The variables included in the analysis were clinical data, such as age, sex, obesity, heart rate (HR), smoking, a history of hypertension and diabetes, and the use of antihypertensive and anti-diabetic medications. The stringent criteria for variable inclusion were set at a kicking boundary value of α = 0.05 for entry into the model, and α = 0.10 for exclusion.

To assess repeatability, κ, linear correlation analysis, and Bland-Altman plots were used, while Pearson correlation analysis was utilized to examine variable correlation.

The relationship between API and its determinants were analyzed by restrictive cubic spline (RCS) with 0th, 5th, 27.5th, 50th, 77.5th, 95th, and 100th percentiles. Statistical analysis of RCS was performed using R version 4.2.2 provided by the R Foundation for Statistical Computing (Vienna, Austria). The construction of chord diagrams was implemented by identifying significantly correlated clinical variables within different groups. The data were analyzed using SPSS 23.0 for Windows (IBM, Armonk, NY, USA). Statistical significance was set as a two-tailed *p*-value < 0.05.

## 3. Results

### 3.1 Baseline Characteristics

**Fig. 1.**
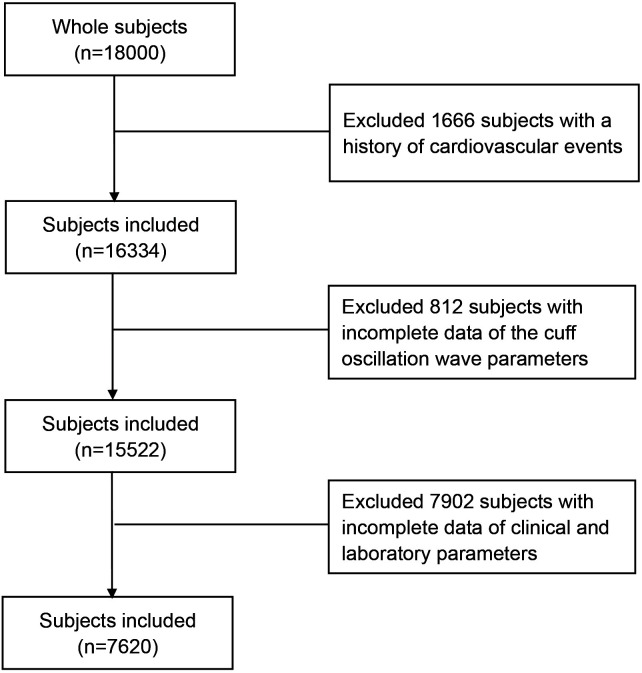
**Flow chart for participant selection with inclusion and exclusion criteria**.

Among the 18,000 participants screened, 7620 participants were enrolled in this study, as depicted in Fig. 1. The demographic comprised 3900 females and 3720 males, with a mean age of 58.46 ± 13.04 years. The study cohort was divided into 3 groups: 1262 participants between the ages of 18–44 years (young adults) with a mean age of 36.35 ± 5.97, in which 645 were female and 617 were male. Next, 2296 participants were between the ages of 45–59 years (middle-aged adults) with a mean age of 53.16 ± 4.31, in which 1275 were female and 1021 were male. Finally, 4062 participants were age ≥60 years (older adults) with a mean age of 68.34 ± 5.68, in which 1980 were female and 2082 were male, as detailed in Table 1.

**Table 1.** **Baseline characteristics of study participants and assessment by age (n = 7620)**.

Characteristics		Young adults (n = 1262)	Middle-aged (n = 2296)	Older adults (n = 4062)	*F/χ^2^* value	*p* value
Demographics						
	Sex, Men, n (%)	617 (48.91)	1021 (44.47)^*^	2082 (51.25)^#^	27.045	<0.001
	Age (years)	36.35 ± 5.97	53.16 ± 4.31^*^	68.34 ± 5.68^*#^	18,767.420	<0.001
	Hypertension, n (%)	453 (35.89)	1238 (53.91)^*^	2719 (66.93)^*#^	401.640	<0.001
	Diabetes, n (%)	125 (9.90)	454 (19.77)^*^	1244 (30.62)^*#^	258.233	<0.001
	Current smoker, n (%)	52 (4.120)	141 (6.14)^*^	476 (11.71)^*#^	97.967	<0.001
	Everyday drinking, n (%)	61 (4.83)	95 (4.13)	324 (7.97)^*#^	42.120	<0.001
Medications						
	Antihypertension, n (%)	86 (8.34)	356 (20.90)^*^	1020 (35.40)^*#^	322.208	<0.001
	Anti-diabetes, n (%)	46 (3.88)	176 (8.72)^*^	509 (15.29)^*#^	132.281	<0.001
Anthropometrics						
	Height (cm)	166.81 ± 8.19	164.45 ± 7.48^*^	163.89 ± 7.88^*#^	67.114	<0.001
	Body mass (kg)	68.19 ± 14.83	66.04 ± 11.66	64.00 ± 10.62^*#^	67.602	<0.001
	BMI (kg/m^2^)	24.34 ± 4.15	24.33 ± 3.35	23.78 ± 3.3^*#^	23.904	<0.001
	SBP (mmHg)	125.95 ± 22.67	135.83 ± 24.42^*^	143.46 ± 27.22^*#^	238.486	<0.001
	DBP (mmHg)	81.62 ± 14.48	84.97 ± 14.93^*^	81.24 ± 14.7^#^	49.469	<0.001
	PP (mmHg)	44.32 ± 14.8	50.86 ± 16.92^*^	62.23 ± 22.4^*#^	500.362	<0.001
	Heart rate (beats/min)	84.00 ± 13.03	79.59 ± 12.82^*^	78.81 ± 12.51^*#^	81.344	<0.001
Biochemical analysis						
	AST (U/L)	33.31 ± 52.18	30.85 ± 34.49^*^	26.05 ± 20.98^*#^	31.606	<0.001
	Uric acid (µmol/L)	329.71 ± 105.06	322.11 ± 93.77^*^	328.26 ± 96.61^#^	3.690	0.025
	Cholesterol (mmol/L)	4.45 ± 0.95	4.64 ± 1.03^*^	4.42 ± 1.08^#^	33.182	<0.001
	HDL (mmol/L)	1.11 ± 0.3	1.14 ± 0.31	1.12 ± 0.32	2.073	0.126
	LDL (mmol/L)	2.86 ± 0.93	2.96 ± 0.94^*^	2.76 ± 1.00^*#^	33.169	<0.001
	FBG (mmol/L)	5.4 ± 1.65	5.83 ± 1.73^*^	6.00 ± 1.84^*#^	55.401	<0.001
	Hemoglobin (g/dL)	137.05 ± 20.8	136.13 ± 18.24	132 ± 17.41^*#^	57.607	<0.001
	Total protein (g/dL)	67.91 ± 6.09	67.32 ± 6.18^*^	65.26 ± 6.27^*#^	130.449	<0.001
	Albumin (g/dL)	45.1 ± 4.49	44.32 ± 4.50^*^	42.53 ± 4.55^*#^	208.676	<0.001
	eGFR (mL/min/1.73 m^2^)	131.79 ± 37.67	121.45 ± 37.81^*^	107.71 ± 34.68^*#^	253.081	<0.001
Arterial stiffness parameters						
	API	26.51 ± 6.11	28.59 ± 6.88^*^	33.08 ± 8.35^*#^	480.469	<0.001
	API ≥31	280 (22.19)	735 (32.01)^*^	2352 (57.90)^*#^	695.506	<0.001

Note: Data are shown as mean ± SD, percent or number (%) of event outcomes. 1 mmHg = 0.133 kPa. BMI, body mass index; SBP, systolic blood pressure; DBP, diastolic blood pressure; PP, pulse pressure; AST, aspartate aminotransferase; HDL, high-density lipoprotein; LDL, low-density lipoprotein; FBG, fasting blood glucose; eGFR, estimated glomerular filtration rate; API, arterial pressure volume index. ^*^Significant different compared to young adults group (*p* < 0.05); ^#^Significant different compared to middle-aged group (*p* < 0.05).

To investigate the impact of overweight/obesity in API, we divided participants into two distinct groups based on a BMI cutoff point of 25. This division was made to establish a clear delineation between an overweight/obese group (BMI of 25 or above) and a control group (BMI below 25). The overweight/obese group had a significantly higher API, as shown in Table 2.

**Table 2.** **Baseline characteristics of study participants and assessment by BMI (n = 7620)**.

Characteristics		Control group (n = 5044)	Overweight/Obese group (n = 2576)	*t/χ^2^* value	*p* value
Demographics					
	Sex, Men, n (%)	2310 (62.1)	1410 (37.9)	54.532	<0.001
	Age (years)	58.97 ± 13.12	57.48 ± 12.83	4.724	<0.001
	Hypertension, n (%)	2659 (52.7)	1751 (68)	162.817	<0.001
	Diabetes, n (%)	1065 (21.1)	758 (29.4)	64.717	<0.001
	Current smoker, n (%)	429 (8.5)	240 (9.3)	1.403	0.236
	Everyday drinking, n (%)	313 (6.2)	167 (6.5)	0.223	0.637
Medications					
	Antihypertension, n (%)	780 (15.5)	682 (26.5)	133.340	<0.001
	Anti-diabetes, n (%)	416 (8.2)	315 (12.2)	31.157	<0.001
Anthropometrics					
	Height (cm)	164.26 ± 7.66	165.1 ± 8.28	–4.442	<0.001
	Body mass (kg)	59.94 ± 7.98	75.83 ± 11.04	–71.878	<0.001
	BMI (kg/m^2^)	22.15 ± 1.94	27.73 ± 2.8	–101.710	<0.001
	SBP (mmHg)	136.41 ± 26.86	141.88 ± 25.32	–8.579	<0.001
	DBP (mmHg)	80.99 ± 14.88	85.24 ± 14.3	–11.934	<0.001
	PP (mmHg)	55.42 ± 21.05	56.65 ± 20.89	–2.417	0.016
	Heart rate (beats/min)	79.97 ± 13.05	79.79 ± 12.37	0.583	0.560
Biochemical analysis					
	AST (U/L)	26.53 ± 39.35	27.66 ± 18.43	–1.389	0.165
	Uric acid (µmol/L)	314.4 ± 95.28	350.64 ± 96.64	–15.630	<0.001
	Cholesterol (mmol/L)	4.48 ± 1.07	4.51 ± 1.01	–1.386	0.166
	HDL (mmol/L)	1.16 ± 0.32	1.05 ± 0.28	15.445	<0.001
	LDL (mmol/L)	2.8 ± 0.98	2.91 ± 0.96	–4.552	<0.001
	FBG (mmol/L)	5.75 ± 1.76	6.05 ± 1.83	–6.882	<0.001
	Hemoglobin (g/dL)	131.88 ± 18.29	138.37 ± 17.84	–14.777	<0.001
	Total protein (g/dL)	66.15 ± 6.42	66.63 ± 6.1	–3.140	0.002
	Albumin (g/dL)	43.31 ± 4.75	43.84 ± 4.43	–4.686	<0.001
	eGFR (mL/min/1.73 m^2^)	115.71 ± 37.82	116.1 ± 36.34	–0.429	0.668
Arterial stiffness parameters					
	API	30.31 ± 8.11	31.27 ± 7.9	–4.911	<0.001
	API ≥31	2115 (41.9)	1252 (48.6)	30.774	<0.001

Note: Data are shown as mean ± SD, percent or number (%) of event outcomes. BMI, body mass index; SBP, systolic blood pressure; DBP, diastolic blood pressure; PP, pulse pressure; AST, aspartate aminotransferase; HDL, high-density lipoprotein; LDL, low-density lipoprotein; FBG, fasting blood glucose; eGFR, estimated glomerular filtration rate; API, arterial pressure volume index.

### 3.2 Reproducibility Analysis

API repeatability experiments demonstrated high consistency, with significant correlations both between groups (R^2^ = 0.786, *p* < 0.001, mean difference –0.07 ± 3.10%) and within groups (R^2^ = 0.735, *p* < 0.001, mean difference –0.58 ± 3.71%). The Bland-Altman analysis confirmed the reliability of API repeat measurements (**Supplementary Fig. 1**). Notably, there was moderate agreement between different observers (κ = 0.885, *p* < 0.001) and repeated measurements by the same observer (κ = 0.855, *p* < 0.001).

### 3.3 Measures of API According to Sex

**Fig. 2.**
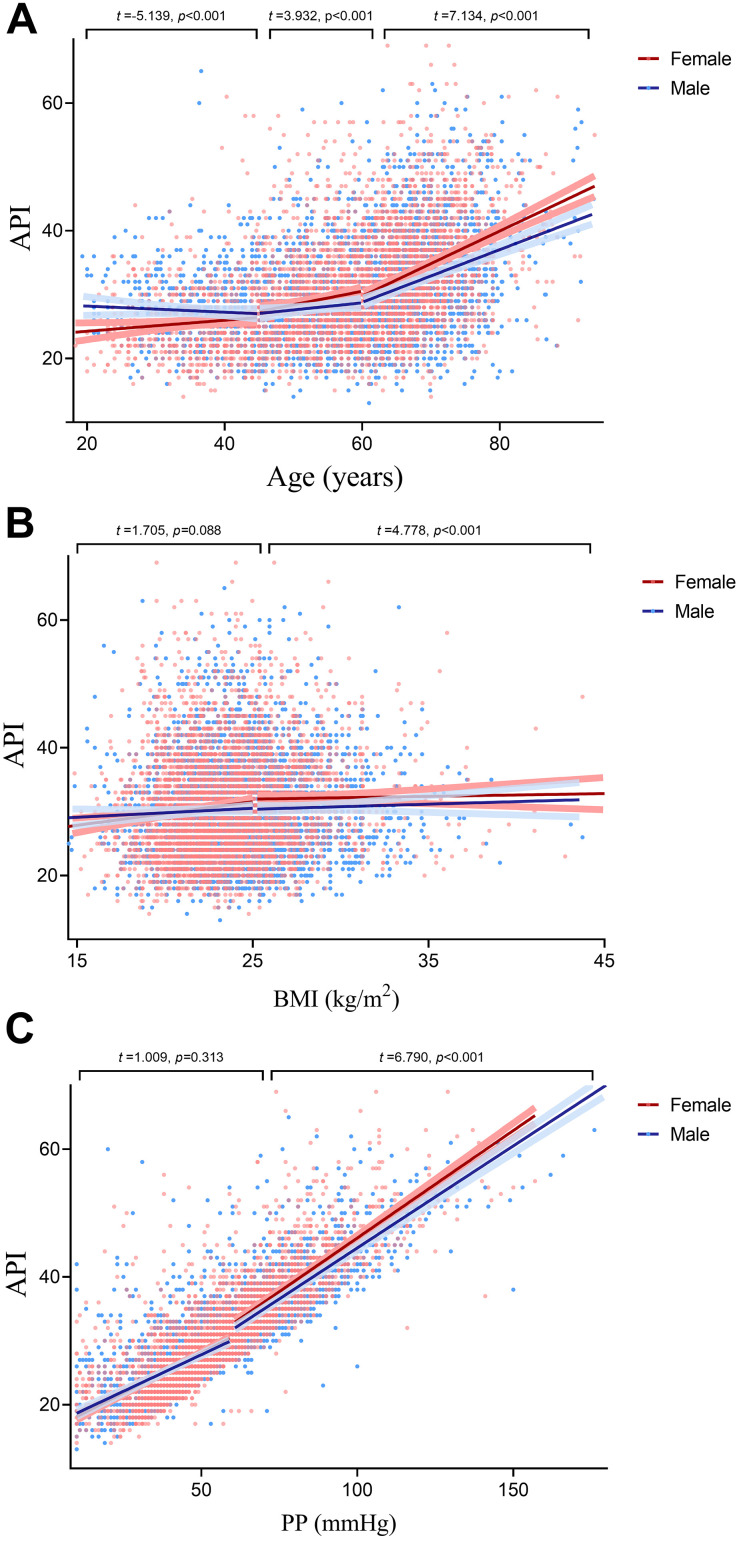
**Linear regression analysis illustrating the correlation between API and age, BMI, and PP**. Analysis of the slopes and intercepts of the two regression lines reveals significant sex differences in API across age (A), BMI (B), and PP (C) groups. BMI, body mass index; API, arterial pressure volume index; PP, pulse pressure.

Scatter plots were utilized to investigate the interaction between sex and age, BMI, or PP in the API (Fig. 2). A clear pattern of sex-based disparities in API were observed across varying age groups and within different BMI and PP groups. Notably, among the young adult demographics, female participants exhibited significantly lower API in comparison to their male counterparts. This trend was reversed, however, within the middle-aged and elderly populations, where female participants recorded notably higher API than males. There was no difference in API between males and females with BMI <25 or PP <60 mmHg. In contrast, females with BMI ≥25 or PP ≥60 mmHg had significantly higher API than males.

### 3.4 Differences in API by Dichotomous Variables

**Fig. 3.**
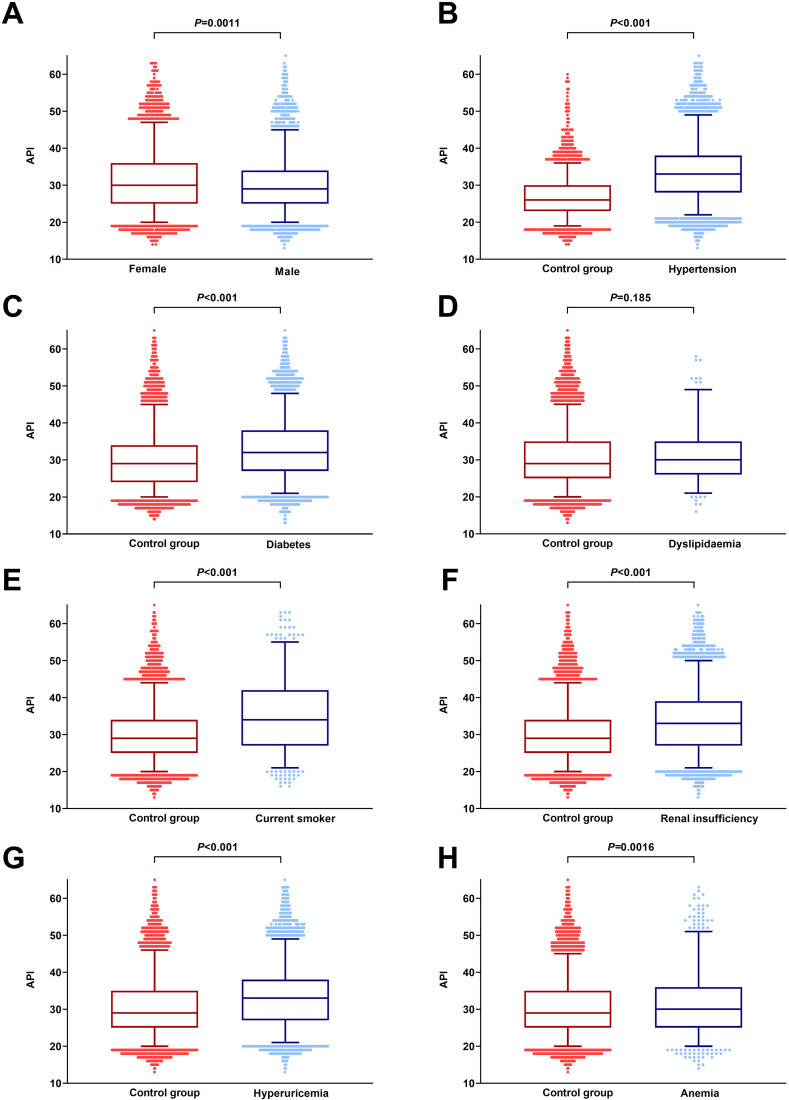
**Distribution of mean API as dichotomous variables, using *t*-tests**. Utilizing a Box-and-whisker plot, we illustrate the dichotomous variables’ impact on API results, of sex (A), hypertension (B), diabetes (C), dyslipidaemia (D), current smoker (E), renal insufficiency (F), hyperuricemia (G), and anemia (H). This graphic depiction encompasses the comprehensive range of data points, starting with the minimal values represented by the lowest horizontal line or dot, and stretching to the maximum values indicated by the highest horizontal line or dot. Additionally, it incorporates the statistical measures of the first quartile, which is denoted by the bottom of the box, the median depicted by the horizontal line within the box, and the third quartile. API, arterial pressure volume index.

The API differences across dichotomous variables are presented in Fig. 3. The analysis revealed that female participants had a significantly higher mean API compared to those of their male counterparts. Additionally, the mean API in participants diagnosed with hypertension, diabetes, current smoker, renal insufficiency, hyperuricemia or anemia was higher compared with those without these co-morbidities.

### 3.5 Relations between API and Clinical Variables

When assessing full cohort, we determined that API was associated with several variables, as shown in Table 3. Correlation analyses revealed strong associations of API with age (*r* = 0.406, *p* < 0.001) and PP (*r* = 0.852, *p* < 0.001). After adjustments for confounding factors, multiple linear regression analysis of API using the stepwise method showed that age, sex, BMI, PP, heart rate, hemoglobin, UA, eGFR, TC and a history of hypertension were each independently associated with API.

**Table 3.** **Association between API and influencing factors of API**.

Item	Simple correlation analysis [*r*]		Multiple linear regression analysis (stepwise) [β]	
	*r*	*p* value	Standardized β-coefficient	*p* value
Sex	–0.030	0.009	–0.049	<0.001
Age (years)	0.406	<0.001	0.055	<0.001
BMI (kg/m^2^)	0.094	<0.001	0.038	<0.001
PP (mmHg)	0.852	<0.001	0.780	<0.001
Heart rate (beats/min)	–0.083	<0.001	–0.050	<0.001
Hemoglobin (g/dL)	–0.080	<0.001	–0.017	0.022
Uric acid (µmol/L)	0.050	<0.001	0.017	0.022
eGFR (mL/min/1.73 m^2^)	–0.154	<0.001	–0.018	0.009
Total cholesterol (mmol/L)	–0.021	0.062	–0.017	0.011
Hypertension	0.442	<0.001	0.052	<0.001

Note: β is the regression coefficient. API, arterial pressure volume index; BMI, body mass index; PP, pulse pressure; eGFR, estimated glomerular filtration rate.

**Fig. 4.**
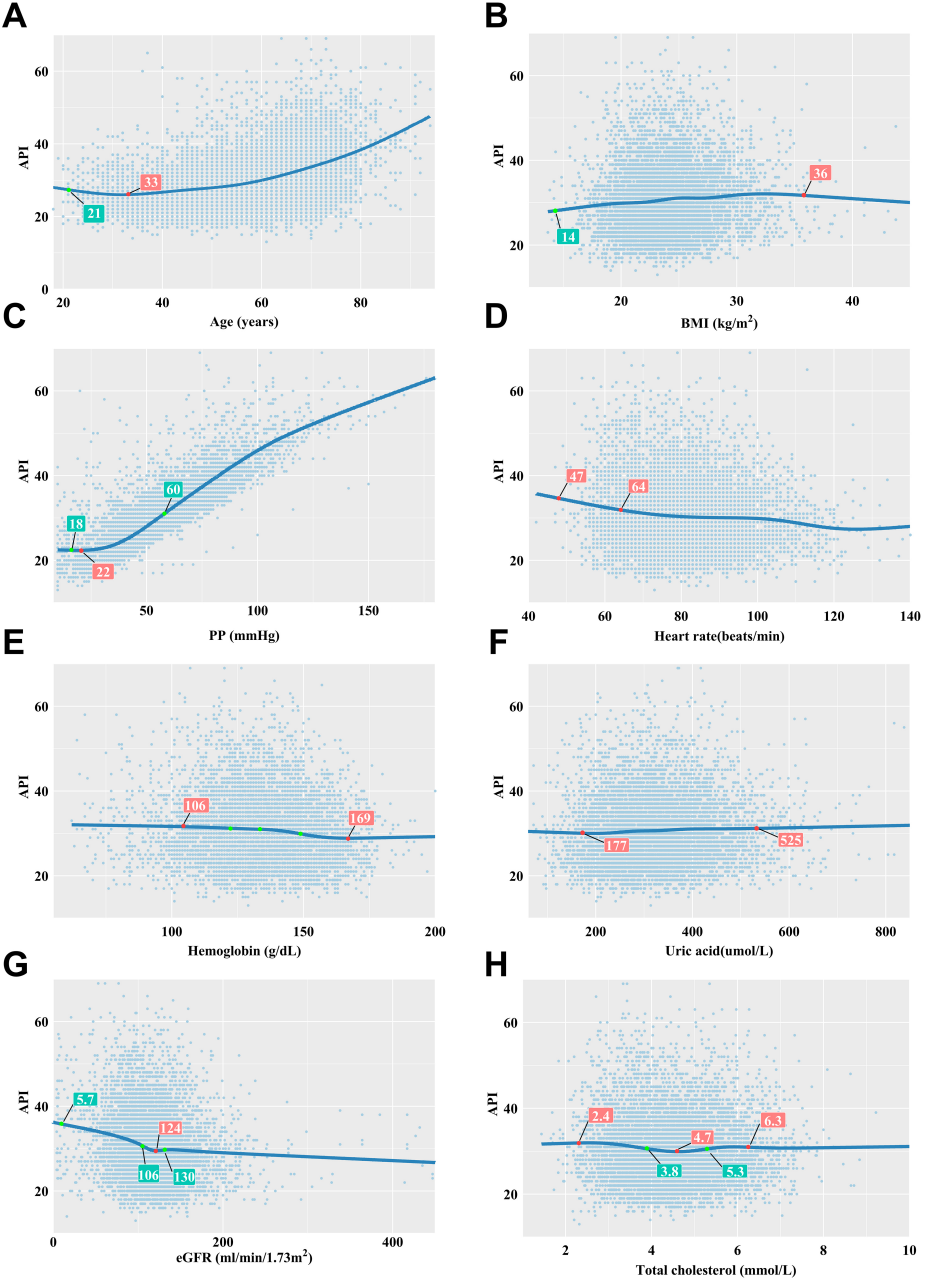
**Correlations between API and its influencing factors based on restricted cubic spline functions**. (A) There was a significant U-shaped relationship between API and age, with 33 years as the age with the lowest API value. (B) There was a significant inverted U-shaped correlation between API and BMI, with the peak API observed at a BMI of 36 kg/m^2^. (C) API increased with PP, notably accelerating at a PP of 60 mmHg. (D) API decreased with increasing heart rate. Particularly rapid API declines were noted at 47 and 64 beats/minute. (E) Higher API levels were present when hemoglobin levels were below 106 units, with a sharp decrease as hemoglobin values exceeded 169 units. (F) As UA levels rose, API showed a moderate increase at 525 units. (G) There was a significant U-shaped relationship between API and eGFR. The minimum API level corresponded to an eGFR of 124 units. (H) The relationship between API and TC was found to be expressed as a U-shaped curve. The TC value corresponding to the lowest API value was 4.7 units. When API increased rapidly, the corresponding TC value was 6.3 units. API, arterial pressure volume index; BMI, body mass index; PP, pulse pressure; eGFR, estimated glomerular filtration rate; UA, uric acid; TC, triglyceride.

Using a restrictive cubic spline for analysis, the relationship between API and several variables was graphically depicted. The analysis unveiled a significant J-shaped dose response relationship between API and age, beginning at 33 years, indicating that API values begin to significantly increase past this age. For PP and UA, there were increases in API, with a rapid increase observed at PP values of 60 mmHg, or UA levels of 525 units. Conversely, an increase of heart rate and eGFR was associated with a decrease in API. Notably, the relationship between API and BMI formed a significant inverted U-shape, as illustrated in Fig. 4.

### 3.6 Risk Factors for High API

The logistic regression model revealed that multiple factors that significantly impacted high API. These included sex, age, obesity, HR, hypertension, diabetes, antihypertensives, current smoker and eGFR, as shown in Table 4.

**Table 4.** **Multivariate logistic regression model analysis of risk factors for high API (stepwise)**.

Item	β	*p* value	OR	95% CI
Overweight	0.219	<0.001	1.245	1.113–1.393
Older adults	1.121	<0.001	3.067	2.745–3.427
High heart rate	–0.368	<0.001	0.692	0.622–0.77
Male	–0.593	<0.001	0.553	0.495–0.617
Hypertension	2.058	<0.001	7.829	6.914–8.864
Diabetes	0.337	<0.001	1.401	1.237–1.586
Antihypertensives	–0.888	<0.001	0.412	0.358–0.473
Current smoker	0.479	<0.001	1.615	1.338–1.95
Renal insufficiency	0.166	0.016	1.181	1.031–1.352

Note: API, arterial pressure volume index; OR, odds ratio; CI, confidence interval.

**Fig. 5.**
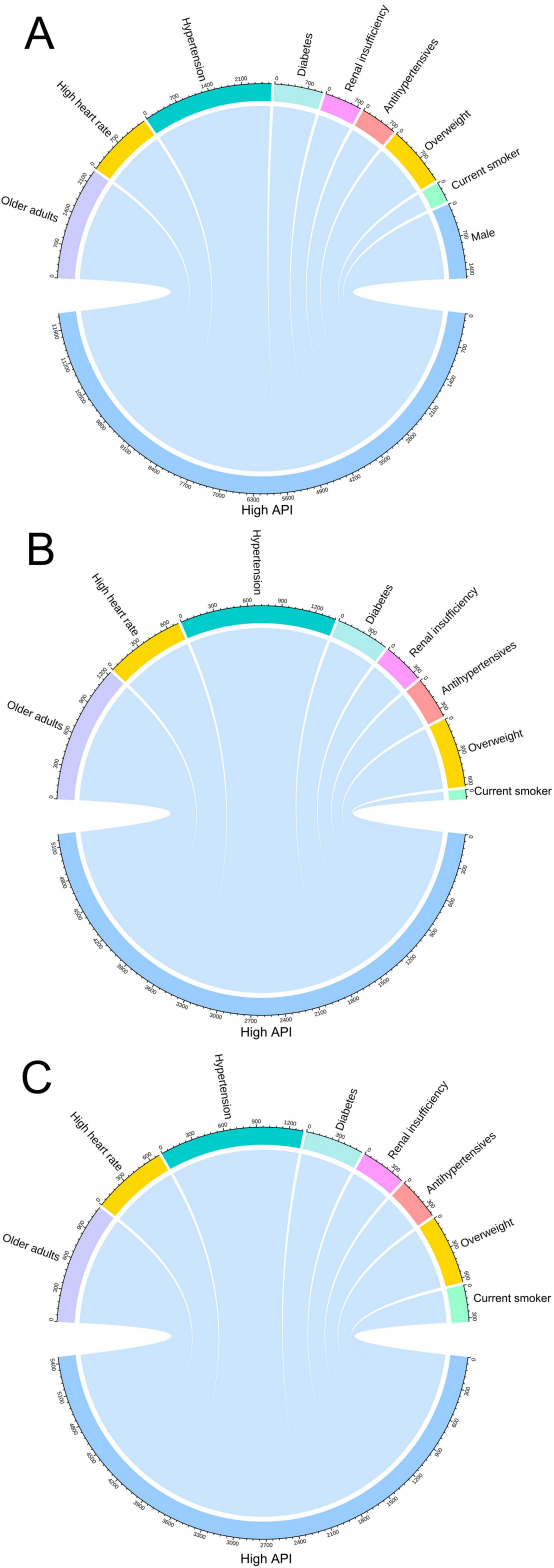
**Visualization of the correlation between high API and abnormal clinical indicators using paired correlation chord diagrams for overall (A), male (B) and female (C)**. The outer colored arcs of the circle represent clinical variables, while the sectors (chords) between them denote significant correlations, with the bandwidth indicating the quantity of related pairs. These diagrams illustrate that the distribution characteristics between high API and abnormal indicators are largely consistent between men and women. High API is mainly associated with older adults and hypertensive participants. API, arterial pressure volume index.

We analyzed the pairwise correlations between high API and abnormal clinical indicators across different groups, utilizing chord diagrams for visualization. These diagrams arrange abnormal clinical indicators around a circle, as shown in Fig. 5. By generating chord plots, we can clearly observe the similarities and differences among the entire group (Fig. 5A), as well as separately among males (Fig. 5B) and females (Fig. 5C). These diagram reveal that the distribution characteristics of high API and abnormal indicators are broadly similar between males and females. High API is mainly associated with older adults and hypertensive participants, followed by obesity and heart rate >80 beats/min (high heart rate).

## 4. Discussion

This study was the first to describe API distribution characteristics within a large cohort from the Chinese population. The findings indicate a positive correlation of API with age and PP. In addition, independent variables such as sex, age, obesity, HR, hypertension, diabetes, use of antihypertensive medication, current smoking status, and eGFR were identified as significant contributors to high API. Furthermore, API displayed a U-shaped relationship with many established CVD risk factors.

Derived from the pressure-vascular volume curvature of the brachial artery, API is calculated through the analysis of oscillatory waves detected by a brachial cuff. This method involves unloading the brachial artery using cuff pressure until it’s nearly stress-free. The reciprocal of coefficient B in the fitting function is referred to as API [17]. Therefore, API reflects the mechanical properties of the brachial artery wall under conditions of zero transmural pressure, and serves as a marker for assess the residual circumferential stresses within the brachial artery [18].

The results of this study support previous findings showing the influence of blood pressure (BP) on API [19]. It further elucidates the distribution residual circumferential stress within the arterial tree: stress decreases along the ascending aorta, spikes at the apex of the aortic arch, then diminishes along the arch, stabilizing near the descending aorta to the diaphragm [20]. This stress pattern again elevates in the abdominal aorta and reaches its peak in the bifurcation of the iliac artery [20]. In addition, studies reported that arterial residual stress is higher at the proximal end than at the distal end, with stress levels varying across the artery wall’s layers [21]. Under normal physiological conditions, these residual stresses help distribute BP evenly along the vessel wall, preventing stress concentration and protecting the intima from pressure-induced damage.

The increase of API increases with age can be attributed to the “stress growth” principle, where unbalanced growth is due to excessive inner layer arterial growth relative to that of the outer wall [22]. In young and middle-aged adults, residual stress increases significantly in the descending and abdominal aorta, whereas in the elderly, significant increases occur in the ascending aorta and aortic arch [23]. This pattern reflects the arterial wall composition: elastin predominates in the proximal aorta, while the distal aortic wall is richer in viscoelastic collagen [21, 24]. Ageing is accompanied by arterial remodeling which ensures the tissue is in constant dynamic equilibrium, these fluctuations influence the biomechanical properties of the arteries [22]. Kamenskiy *et al*. [25] conducted arterial biomechanical analyses from different age groups. Their group demonstrated that aging shifts stress concentration areas in the vessel wall—from predominantly intima in youth, to a more even distribution across the wall in middle age, and finally to the adventitia area in old age [25]. The observed changes in API could be attributed to alterations in the composition or configuration of the vascular wall tissue that occur with age.

This study identified sex, HR, BMI and eGFR as independent factors that influence API. It was possible that HR induced changes in vascular structure and function through cellular mechanical receptors, which in turn influence vessel viscoelasticity and circumferential residual stress [26, 27]. The sex difference in API could be attributed to accelerated arteriosclerosis in postmenopausal women through decreased estrogen levels, which induces a variety of effects including oxidative stress, iron accumulation [21, 28], lipid metabolism disruption, and impaired vascular endothelial function [29]. Additionally, obesity exacerbates oxidative stress and vascular inflammation [30], impairing vascular endothelial function, which leads to vascular remodeling and in an increase in circumferential residual stress within the blood vessels [31]. This process, in turn, can contribute to kidney injury through elevated arterial stiffness, resulting in heightened circumferential and shear stresses within the arterial lumen [32]. These hemodynamic stresses exerted on the renal vasculature may lead to endothelial dysfunction and microvascular ischemia, further exacerbating kidney damage [33]. Our previous research has demonstrated the link between API and CVD in the Chinese population (China-PAR) [34]. This study adds to the body of knowledge by uncovering potential age- and overweight -related variations in API, offering valuable insights for assessing residual stress changes across different cardiovascular diseases.

## 5. Limitations

This study does have certain limitations. First, as a single-center cross-sectional retrospective study on a cohort from the Chinese population, it relied on linear regression analysis to identify independent API risk factors. Second, it lacked outcome events related to brachial artery stiffness for reference. Third, the residual stress of the artery is anisotropic and can be categorized into circumferential and axial directions [4]. While the API assesses circumferential residual stresses of the brachial artery, the axial residual stress needs to be investigated in future prospective studies. Fourth, the study design initially required API to be measured twice at different times and dates. However, some participants declined repeat assessments due to long waiting times, potentially introducing measurement bias to the study. Traditionally, measuring residual stress was an invasive procedure restricted to clinical settings; thus, API offers a novel, non-invasive alternative for evaluating residual stress. Future research in our laboratory will expand on the measurement of residual stress techniques.

## 6. Conclusions

This study was the first to describe API distribution characteristics in a large sample of the Chinese population. There was a U-shaped relationship between API and age, which was closely related to traditional CVD factors. Overall, API can provide a new perspective for non-invasive assessment of residual stress vascular diseases.

## Data Availability

All data generated or used during the study appear in the submitted article.

## Supplementary Material

Supplementary material associated with this article can be found, in the online version, at https://doi.org/10.31083/j.rcm2508289.
