# Supplementary material for: Unraveling Changes of Brachial Artery Residual Stress and Its Relationship to Cardiovascular Disease Risk Factors
Source: Rev Cardiovasc Med. 2024 Aug 16;25(8):289. doi: 10.31083/j.rcm2508289 (PMC11366995; doi:10.31083/j.rcm2508289)
Supplement: Supplementary file 1 [file 2153-8174-25-8-289-s1.docx]

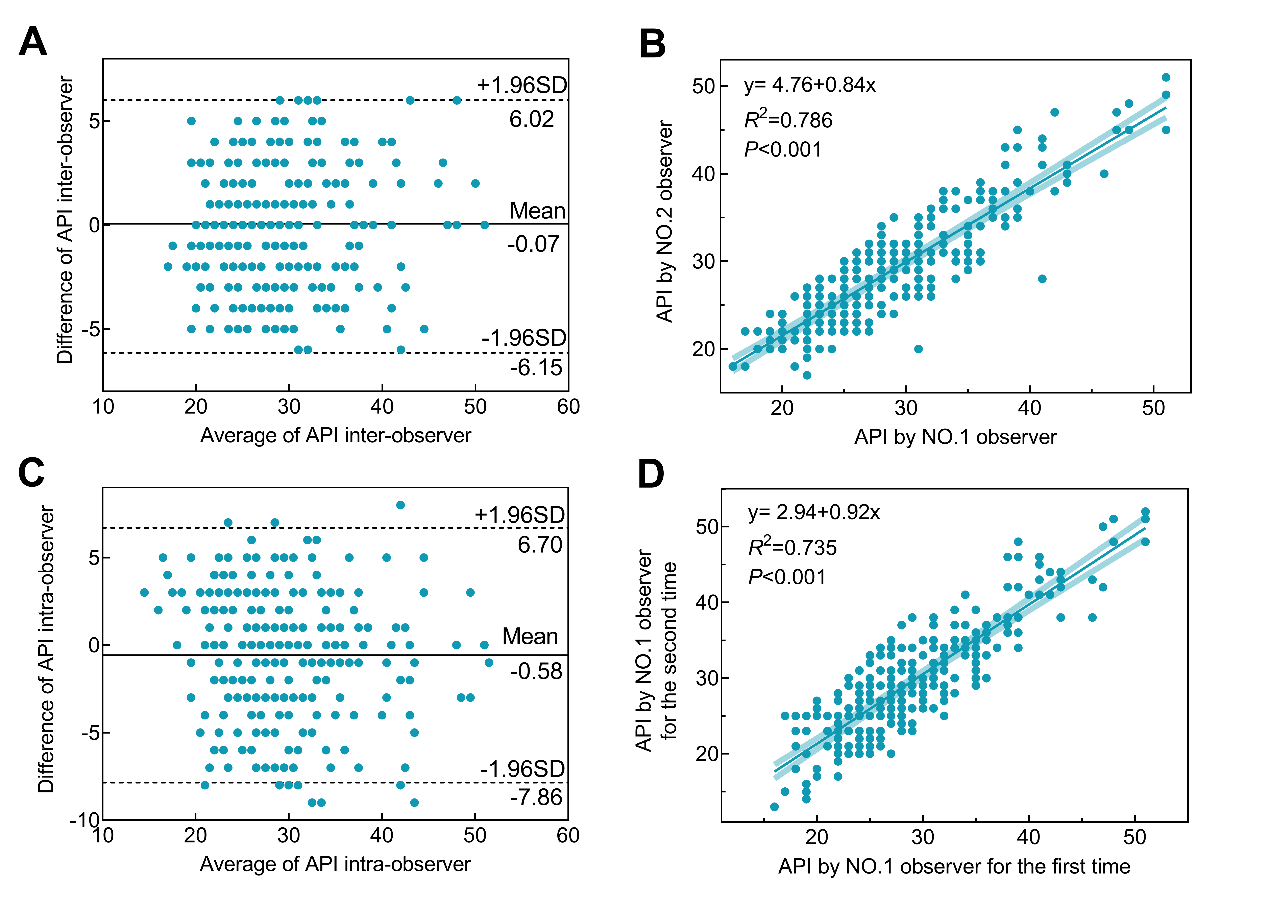


**Supplementary Fig. 1. Repeatability test of API by Bland-Altman plot and linear correlation analysis.** Bland-Altman analysis (**A** and **C**) showed a consistent trend and linear regression analysis (**B** and **D**) showed good agreement for API inter-observer and intra-observer respectively. API, arterial pressure volume index.
